# Post-hoc analysis of the safety and efficacy of isavuconazole in older patients with invasive fungal disease from the VITAL and SECURE studies

**DOI:** 10.1038/s41598-023-31788-1

**Published:** 2023-04-25

**Authors:** Kamal Hamed, Marc Engelhardt, Laura L. Kovanda, Jin Ju Huang, Jean Yan, Jalal A. Aram

**Affiliations:** 1 Basilea Pharmaceutica International Ltd., Allschwil, Switzerland; 2 Astellas Pharma Global Development, Inc., Northbrook, IL USA; 3 Pfizer, 10645848 PBG China Medical, Beijing, China; 4 Pfizer Inc., New York, USA

**Keywords:** Infectious diseases, Fungi

## Abstract

Isavuconazole is a triazole with broad-spectrum antifungal activity. In this post-hoc analysis of two prospective clinical trials (VITAL and SECURE), the safety and efficacy of isavuconazole in patients aged ≥ 65 years with invasive fungal diseases were evaluated. Patients were divided into two subgroups (≥ 65 and < 65 years). Adverse events (AEs); all-cause mortality; and overall, clinical, mycological, and radiological response were assessed. A total of 155 patients ≥ 65 years were enrolled in both trials. Most patients reported AEs. In the isavuconazole arm of both studies, serious AEs (SAEs) were greater in patients ≥ 65 versus < 65 years: 76.7% versus 56.9% (VITAL); 61.9% versus 49.0% (SECURE). In SECURE, SAE rates were similar in the ≥ 65 years subgroup of both treatment arms (61.9% vs 58.1%), while in the < 65 years subgroup the SAE rate was lower in the isavuconazole arm (49.0% vs 57.4%). In VITAL, all-cause mortality through day 42 (30.0% vs 13.8%) was higher, and overall response at end of treatment (27.6% vs 46.8%) was lower in patients ≥ 65 years versus < 65 years. In SECURE, all-cause mortality was similar between both subgroups, and isavuconazole (20.6% vs 17.9%) and voriconazole (22.6% vs 19.4%) treatment arms. The overall response was lower in the ≥ 65 years than the < 65 years subgroup in the isavuconazole (23.7% vs 39.0%) and voriconazole (32.0% vs 37.5%) arms. The safety and efficacy of isavuconazole were better in patients < 65 versus ≥ 65 years, and the safety profile was more favorable than that of voriconazole in both subgroups.

*Clinicaltrials.gov identifier* NCT00634049 and NCT00412893.

## Introduction

Invasive fungal diseases (IFDs), such as invasive mucormycosis (IM) and invasive aspergillosis (IA), are life-threatening infections that remain a major cause of morbidity and mortality in a wide range of at-risk and immunocompromised populations such as those with hematological malignancies, organ transplant, individuals on prolonged immunosuppressive therapy, and older individuals^1–3^. *Candida albicans*, *Aspergillus fumigatus*, *Cryptococcus neoformans*, *Pneumocystis jirovecii*, endemic dimorphic fungi, and fungi of the Mucorales order are the etiologic agents responsible for most cases of IFD globally^1,4–6^. The estimated global annual incidence for IM and IA is > 10,000 cases and > 300,000 cases^4^, with mortality rates of 35–96%^7^ and 30–70%^4,8, 9^, respectively. Older individuals are disproportionately affected by IFDs, and, despite the availability of effective treatment options, may experience worse disease outcomes and higher mortality rates than younger individuals^1,3^.

During the severe acute respiratory syndrome coronavirus 2 (SARS-CoV-2) pandemic, a rise in the proportion of older patients with IFDs was observed^10–12^. IFDs caused by primary and opportunistic pathogens are identified among older individuals with immunosenescence^13^. Age is a significant predisposing factor for the development of fungal infection and is associated with increased mortality^14–18^. Comorbidities, increased use of corticosteroids and immunosuppressants, drug-drug interactions (DDIs) associated with polypharmacy, age-related changes in organ function, and a higher risk of adverse drug reactions, could make older individuals more vulnerable to fungal infections and the clinical management of their IFDs more challenging^19,20^.

As the pace of population aging increases worldwide, the proportion of individuals aged over 60 years is estimated to double (from 12 to 22%) between 2015 and 2050^21,22^. A gap in understanding the safety and efficacy profile of antifungal drugs in older individuals exists since randomized controlled trials are mainly focused on the overall population^19^. Therefore, there is a growing need for safe and effective therapeutic options for IFDs in this population.

Isavuconazole, the active moiety of the water-soluble prodrug isavuconazonium sulfate, is a broad-spectrum triazole approved by the US Food and Drug Administration and the European Medicines Agency for the treatment of IA and IM in adults^23,24^. For the optimal use of isavuconazole in older individuals, it is essential to understand its safety and efficacy profile in this age group. To this end, we conducted a post-hoc analysis of two prospective clinical trials to evaluate the safety and efficacy of isavuconazole in patients with IFDs aged ≥ 65 years.

## Methods

### Study design

**Figure 1:**
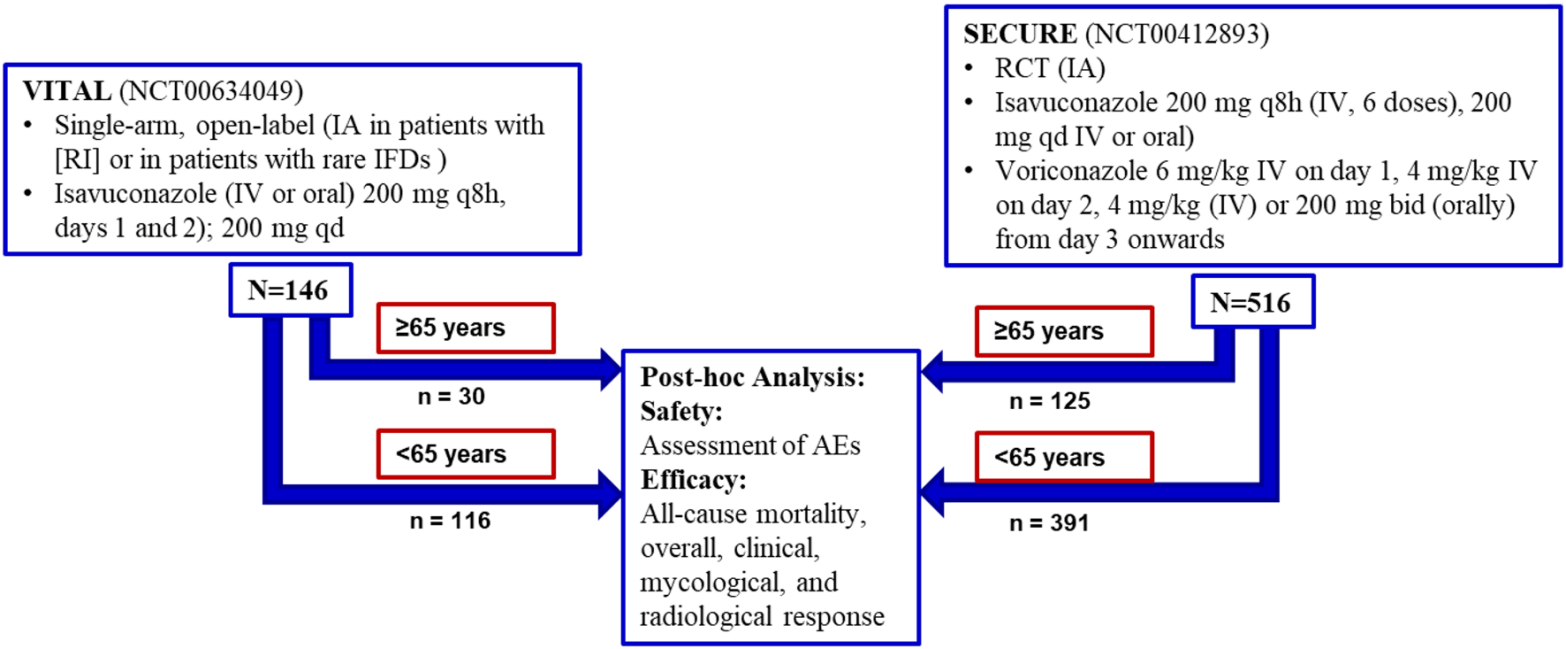
Description of the studies used in this post-hoc analysis and study design. AEs = adverse events, bid = twice a day, IFD = Invasive fungal disease; IA = invasive aspergillosis, IV = Intravenous, q8h = every 8 h, qd = once a day, RCT = randomized controlled trial, RI = Renal impairment.

Data from two prospective clinical trials, VITAL (NCT00634049, completed in 2016) and SECURE (NCT00412893, completed in 2013), were analyzed for patients aged ≥ 65 and < 65 years (Fig. 1)^25,26^. Both studies were conducted in compliance with the Declaration of Helsinki and the International Conference on Harmonization Good Clinical Practice Guidelines (ICH-GCP). The final protocols and amendments were reviewed and approved by the Institutional Review Boards and Independent Ethics Committees of the investigational centers at participating sites and details are provided in Supplementary Information. Patients provided written informed consent. The study designs of these two trials have been published previously^25,26^ and are summarized below.

### VITAL study

This was a single-arm, open-label study conducted to evaluate isavuconazole in the treatment of IA in patients with renal impairment (defined as calculated creatinine clearance [CLCr] < 50 mL/min at enrollment) requiring primary therapy or in patients with rare IFDs including patients with mucormycosis (primary therapy or refractory or intolerant to other therapies) or patients infected with rare yeasts or dimorphic fungi (i.e., fungal pathogens other than *Aspergillus fumigatus* or *Candida* species) with or without renal impairment. Patients were required to meet the criteria for proven, probable, or possible IFD described by the European Organization for the Research and Treatment of Cancer/Mycoses Study Group (EORTC/MSG) criteria^27,28^. A total of 146 adult patients from 34 centers worldwide were treated with isavuconazole.

Patients received six doses of either oral or intravenous (IV) isavuconazole 200 mg (administered as isavuconazonium sulfate 372 mg) every 8 h, followed by a once-daily dose until resolution of IFD or for a maximum of 180 days.

### SECURE study

This randomized, double-blind, non-inferiority study that compared isavuconazole with voriconazole for the treatment of IA was conducted at 102 centers across 26 countries worldwide. Overall, 516 adult patients were randomized (1:1) and treated (258 per group). Patients who met the criteria for proven, probable, or possible IFD caused by *Aspergillus* species or other filamentous fungi based on EORTC/MSG guideline were eligible for enrollment^28^.

Patients were randomized to receive either isavuconazole or voriconazole. Isavuconazole 200 mg (i.e., isavuconazonium sulfate 372 mg) was administered IV every 8 h on days 1 and 2, followed by either IV or oral isavuconazole 200 mg once daily. Voriconazole 6 mg/kg was administered IV twice daily on day 1, followed by 4 mg/kg IV twice daily on day 2, and 4 mg/kg IV or 200 mg twice daily orally from day 3 onwards. Placebo was used to maintain blinding by matching the frequency of daily dosing. The maximum treatment duration was 84 days.

### Post-hoc analysis

In this post-hoc analysis (performed between 2020 and 2021), patients were divided into subgroups by age, ≥ 65 years and < 65 years, and the safety and efficacy of isavuconazole was evaluated alone (VITAL) or compared to voriconazole (SECURE). Safety was assessed based on incidence and type of adverse events (AEs), classified by system organ class (SOC), in the safety population, which consisted of all randomized patients who received ≥ 1 dose of the study drug. Patient subgroups were compared descriptively.

Efficacy (all-cause mortality through day 42 and data review committee [DRC]-assessed overall, clinical, mycological, and radiological response at day 42 and end of treatment [EOT]) was assessed in the intent-to-treat (ITT, included all patients who received ≥ 1 dose of study drug) and modified ITT (mITT, which consisted of ITT patients with proven or probable IFD, as determined by the DRC) populations. For the analysis of all-cause mortality, crude mortality rate, and for all other responses, the crude success rate was calculated within each treatment group. The 95% confidence interval (CI) for the treatment group was based on a binomial distribution. All endpoints were analyzed descriptively. Data are presented separately for subgroups of patients aged ≥ 65 years and < 65 years for both studies.

### Ethical approval and consent to participate

Both studies included in this post-hoc analysis were conducted in compliance with the Declaration of Helsinki and the International Conference on Harmonization Good Clinical Practice Guidelines (ICH-GCP). The final protocols and amendments were reviewed and approved by the Institutional Review Boards and Independent Ethics Committees of the investigational centers. Written informed consent was obtained from the patients.

### Presentation

The results of these studies were presented in part as an abstract and poster at The American Society of Hematology (ASH) meeting held virtually on November 5, 2021^29^.

## Results

Most patients included in this analysis from the VITAL and SECURE studies were < 65 years at study entry. Of 146 patients in VITAL, 30 were ≥ 65 years and 116 were < 65 years, and of 516 patients in SECURE, 125 were ≥ 65 years and 391 were < 65 years and were included in this analysis.

### Patient disposition and baseline characteristics

In VITAL, when categorized based on age at study entry (≥ 65 years [n = 30]; < 65 years [n = 116]), other baseline characteristics between both subgroups were largely comparable. The proportion of patients with renal impairment (defined as estimated glomerular filtration rate < 60 mL/min/1.73m^2^) was higher in the ≥ 65 years subgroup (56.7%) compared to the < 65 years subgroup (36.5%). Most patients had proven IFD in both subgroups (≥ 65 years: 73.3%, < 65 years: 79.3%). Among all high-risk factors, uncontrolled malignancy was more prevalent in patients aged ≥ 65 years (43.3%) than in those aged < 65 years (28.4%) (Table 1). The most common underlying disease was chronic lymphocytic leukemia in the ≥ 65 years subgroup (16.7%) and acute myeloid leukemia in the < 65 years subgroup (21.6%) (Table 2).

**Table 1:** Demographic and baseline characteristics (safety population).

Parameter	VITAL study		SECURE study			
	Isavuconazole^a^		Isavuconazole^a^		Voriconazole^b^	
	≥ 65 years n = 30	< 65 years n = 116	≥ 65 years n = 63	< 65 years n = 194	≥ 65 years n = 62	< 65 years n = 197
Age, years, mean (SD)	72.5 (6.4)	44 (13.3)	70.2 (4.4)	44.9 (13.5)	70.5 (4.4)	45.1 (12.9)
Median (min–max)	70.5 (65–92)	45.5 (18–64)	69.0 (65–82)	47 (17–64)	69.0 (65–87)	45 (18–64)
Gender, Men, n (%)	23 (76.7)	77 (66.4)	40 (63.5)	105 (54.1)	41 (66.1)	122 (61.9)
Race, n (%)						
White	24 (80.0)	84 (72.4)	57 (90.5)	154 (79.4)	57 (91.9)	134 (68.4)
Asian	3 (10.0)	21 (18.1)	0	38 (19.6)	0	60 (30.6)
Black or African American	2 (6.7)	8 (6.9)	6 (9.5)	1 (0.5)	5 (8.1)	1 (0.5)
Other	1 (3.3)	3 (2.6)	0	1 (0.5)	0	1 (0.5)
Ethnicity, n (%)						
Hispanic or Latino	3 (10.0)	19 (16.4)	0	22 (11.3)	2 (3.2)	7 (3.6)
Not Hispanic or Latino	27 (90.0)	97 (83.6)	63 (100)	172 (88.7)	60 (96.8)	189 (96.4)
BMI (kg/m^2^), mean (SD)	24.67 (5.05)	24.32 (5.90)	24.73 (4.13)	189 (5.26)	24.98 (4.01)	19.2 (4.62)
eGFR-MDRD category (mL/min/1.73m^2^), n (%)						
< 60	17 (56.7)	42 (36.5)	10 (16.4)	10 (5.3)	13 (21.3)	20 (10.5)
≥ 60	13 (43.3)	73 (63.6)	51 (83.6)	179 (94.7)	48 (78.7)	170 (89.5)
Missing	0	1	2	5	1	7
Certainty of diagnosis for IFD^c^ n (%)						
Proven	22 (73.3)	92 (79.3)	9 (14.3)	20 (10.3)	9 (14.5)	27 (13.8)
Probable	7 (23.3)	19 (16.4)	29 (46.0)	85 (43.6)	16 (25.8)	77 (39.3)
Possible	1 (3.3)	2 (1.7)	21 (33.3)	67 (34.4)	28 (45.2)	80 (40.8)
No	0	3 (2.6)	4 (6.3)	23 (11.8)	9 (14.5)	12 (6.1)
Common risk factors at baseline, n (%)						
Hematologic malignancies	14 (46.7)	49 (42.2)	53 (84.1)	158 (81.4)	44 (71.0)	178 (90.4)
Uncontrolled malignancies	13 (43.3)	33 (28.4)	49 (77.8)	124 (63.9)	44 (71.0)	143 (72.6)
Neutropenic	8 (26.7)	30 (25.9)	41 (65.1)	122 (62.9)	40 (64.5)	135 (68.5)
Use of T-cell immunosuppressant	12 (40.0)	49 (42.2)	25 (39.7)	86 (44.3)	27 (43.5)	82 (41.6)
Use of corticosteroid	9 (30.0)	26 (22.4)	11 (17.5)	37 (19.1)	10 (16.1)	29 (14.7)
Allogeneic BMT status	1 (3.3)	25 (21.6)	7 (11.1)	47 (24.2)	3 (4.8)	48 (24.4)

Allogeneic BMT status was defined as Yes for patients who were noted as having a BMT and Allogeneic for type of transplant on the primary underlying disease or condition. Uncontrolled malignancy status was defined as Yes for patients who had a malignancy diagnosis and New diagnosis/Active disease/Relapse present.

*BMI* body mass index, *BMT* bone marrow transplant, *DRC* data review committee, *eGFR-MDRD* estimated glomerular filtration rate calculated using the modification of diet in renal disease formula, *SD* standard deviation, *n* number of patients.

^a^Patients received either oral or intravenous isavuconazole 200 mg every 8 h for six doses, followed by a once-daily dose.

^b^Voriconazole was administered intravenously as 6 mg/kg twice daily on day 1, 4 mg/kg twice daily on day 2, and 4 mg/kg or orally as 200 mg twice daily from day 3 onwards.

^c^Categorized as per the IFD assessment by the independent DRC.

**Table 2:** Primary underlying disease or condition (safety population).

Parameter	VITAL study		SECURE study			
	Isavuconazole		Isavuconazole		Voriconazole	
	≥ 65 years n = 30	< 65 years n = 116	≥ 65 years n = 63	< 65 years n = 194	≥ 65 years n = 62	< 65 years n = 197
Number of patients with malignancy, n (%)	18 (60.0)	51 (44.0)	55 (87.3)	162 (83.1)	46 (74.2)	182 (92.9)
New Diagnosis/Active Disease	11 (36.7)	22 (19.0)	39 (61.9)	83 (42.6)	37 (59.7)	104 (53.1)
Relapse	2 (6.7)	11 (9.5)	10 (15.9)	41 (21.0)	7 (11.3)	39 (19.9)
Remission	5 (16.7)	18 (15.5)	6 (9.5)	38 (19.5)	2 (3.2)	39 (19.9)
Primary underlying disease, n (%)	28 (93.3)	93 (80.2)	63 (100.0)	190 (97.4)	61 (98.4)	194 (99.0)
Acute myeloid leukemia	4 (13.3)	25 (21.6)	28 (44.4)	71 (36.4)	30 (48.4)	96 (49.0)
Acute lymphocytic leukemia	0	8 (6.9)	1 (1.6)	29 (14.9)	4 (6.5)	22 (11.2)
Chronic lymphocytic leukemia	5 (16.7)	4 (3.4)	6 (9.5)	4 (2.1)	4 (6.5)	9 (4.6)
Myelodysplastic syndrome	1 (3.3)	0	5 (7.9)	2 (1.0)	0	8 (4.1)
Refractory anemia with an excess of blasts	1 (3.3)	2 (1.7)	4 (6.3)	11 (5.6)	1 (1.6)	3 (1.5)
Non-Hodgkin’s lymphoma	0	2 (1.7)	3 (4.8)	15 (7.7)	3 (4.8)	5 (2.6)
Diabetes mellitus	2 (6.7)	7 (6.0)	2 (3.2)	2 (1.0)	0	0
B-cell lymphoma	0	1 (0.9)	2 (3.2)	2 (1.0)	2 (3.2)	2 (1.0)
Paraneoplastic pemphigus	0	0	2 (3.2)	0	0	0
Chronic obstructive pulmonary disease	2 (6.7)	5 (4.3)	0	5 (2.6)	3 (4.8)	0
Aplastic anemia	0	4 (3.4)	1 (1.6)	8 (4.1)	4 (6.5)	3 (1.5)

Primary underlying disease in ≥ 3.2% of the patients in at least one of the studies are listed.

*n* number of patients.

The median total duration for isavuconazole treatment was 61 (range: 2–509) days with 6.5 (range: 0.5–37) days of IV dosing and 80 (range: 1.5–501) days of oral dosing in the ≥ 65 years subgroup; and 122 (range: 1–882) days with 10.5 (range: 1–77) days of IV dosing and 140 (range: 3–882) days of oral dosing in the < 65 years subgroup.

In SECURE, the number of patients aged ≥ 65 years and those < 65 years were nearly equal between both treatment arms. At baseline, most parameters were comparable between the ≥ 65 years and < 65 years subgroups in the isavuconazole and voriconazole treatment arms (Table 1). Among all high-risk factors, uncontrolled malignancy status was more prevalent in the ≥ 65 years subgroup (77.8%) than the < 65 years (63.9%) subgroup in the isavuconazole arm, although hematologic malignancy was more prevalent in the < 65 years subgroup (90.4%) than the ≥ 65 years subgroup (71.0%) in the voriconazole arm. At baseline, most patients had probable or possible IFD in both subgroups of both treatment arms (Table 1). The most common underlying disease was acute myeloid leukemia in both subgroups of both treatment arms (Table 2).

The median treatment duration (total dosing) for isavuconazole was 43 (range: 3–87) days with IV dosing for 6 (range: 2–26) days and oral dosing for 60 (range: 0.5–83) days in the ≥ 65 years subgroup; 45 (range: 1–102) days with 4.5 (range: 1–84) days of IV dosing and 60 (range: 0.5–99.5) days of oral dosing in the < 65 years subgroup; and was similar to that for voriconazole in both subgroups.

### Safety

In VITAL, similar proportions of patients with AEs were reported in both subgroups (≥ 65 years: 100%, < 65 years: 94%) after treatment with isavuconazole; with infections and infestations (≥ 65 years: 63.3%, < 65 years: 62.1%) being the most common (Tables 3 and 4). However, a higher proportion of nervous system disorders (≥ 65 years: 50%, < 65 years: 30.2%), psychiatric disorders (≥ 65 years: 43.3%, < 65 years: 19.0%), and cardiac disorders (≥ 65 years: 30.0%, < 65 years: 13.8%) were reported in the ≥ 65 years subgroup (Table 4). The proportion of patients with AEs of mild and moderate intensity was lower and severe intensity was higher in the ≥ 65 years subgroup (mild: 10%, moderate: 23.3%, severe: 66.7%) than the < 65 years subgroup (mild: 19%, moderate: 30.2%, severe: 44.8%).

**Table 3:** Overview of treatment-emergent adverse events and death (safety population).

Parameter, n (%)	VITAL study		SECURE study			
	Isavuconazole		Isavuconazole		Voriconazole	
	≥ 65 years n = 30	< 65 years n = 116	≥ 65 years n = 63	< 65 years n = 194	≥ 65 years n = 62	< 65 years n = 197
AEs	30 (100.0)	109 (94.0)	62 (98.4)	185 (95.4)	62 (100.0)	193 (98.0)
Drug-related AEs	10 (33.3)	50 (43.1)	28 (44.4)	81 (41.8)	34 (54.8)	121 (61.4)
AEs leading to permanent discontinuation of study drug	4 (13.3)	15 (12.9)	8 (12.7)	29 (14.9)	17 (27.4)	42 (21.3)
AEs leading to death	17 (56.7)	27 (23.3)	17 (27.0)	45 (23.2)	19 (30.6)	53 (26.9)
Drug-related AEs leading to permanent discontinuation of study drug	0	7 (6.0)	5 (7.9)	16 (8.2)	9 (14.5)	26 (13.2)
Drug-related AEs leading to death	0	1 (0.9)	1 (1.6)	6 (3.1)	0	6 (3.0)
Deaths^a^	18 (60.0)	29 (25.0)	25 (39.7)	56 (28.9)	24 (38.7)	63 (32.0)
Deaths through 28 days after the last dose of study drug	17 (56.7)	25 (21.6)	17 (27.0)	45 (23.2)	19 (30.6)	51 (25.9)
Serious AEs	23 (76.7)	66 (56.9)	39 (61.9)	95 (49.0)	36 (58.1)	113 (57.4)
Drug-related SAEs	2 (6.7)	11 (9.5)	7 (11.1)	21 (10.8)	4 (6.5)	25 (12.7)

Treatment-emergent AE is an AE starting after first study drug administration until 28 days after the last dose of study drug. Study drug-related AEs include those reported as remotely, possibly, or probably related to the study medication by the investigator and those with a missing relationship. An AE with a missing seriousness assessment was considered as serious.

*AEs* adverse events, *SAEs* serious adverse events.

^a^All reported deaths after first dose of study drug are summarized, regardless of the number of study days after the last dose of study drug.

**Table 4:** Treatment-emergent adverse events by system organ class (safety population).

System organ class)^a^, n (%)	VITAL study		SECURE study			
	Isavuconazole		Isavuconazole		Voriconazole	
	≥ 65 years n = 30	< 65 years n = 116	≥ 65 years n = 63	< 65 years n = 194	≥ 65 years n = 62	< 65 years n = 197
Overall^b^	30 (100.0)	109 (94.0)	62 (98.4)	185 (95.4)	62 (100.0)	193 (98.0)
Gastrointestinal disorders	18 (60.0)	65 (56.0)	46 (73.0)	128 (66.0)	40 (64.5)	140 (71.1)
Infections and infestations	19 (63.3)	72 (62.1)	44 (69.8)	108 (55.7)	34 (54.8)	124 (62.9)
General disorders and administration site conditions	18 (60)	50 (43.1)	38 (60.3)	110 (56.7)	38 (61.3)	106 (53.8)
Respiratory, thoracic, and mediastinal disorders	11 (36.7)	52 (44.8)	34 (54.0)	109 (56.2)	36 (58.1)	111 (56.3)
Metabolism and nutrition disorders	16 (53.3)	46 (39.7)	29 (46.0)	79 (40.7)	28 (45.2)	93 (47.2)
Nervous system disorders	15 (50.0)	35 (30.2)	27 (42.9)	68 (35.1)	24 (38.7)	65 (33.0)
Skin and subcutaneous tissue disorders	8 (26.7)	31 (26.7)	26 (41.3)	60 (30.9)	22 (35.5)	88 (44.7)
Investigations (abnormal laboratory tests)	6 (20.0)	28 (24.1)	22 (34.9)	63 (32.5)	19 (30.6)	77 (39.1)
Blood and lymphatic system disorders	4 (13.3)	25 (21.6)	21 (33.3)	56 (28.9)	13 (21.0)	69 (35.0)
Psychiatric disorders	13 (43.3)	22 (19.0)	22 (34.9)	48 (24.7)	29 (46.8)	57 (28.9)
Musculoskeletal and connective tissue disorders	8 (26.7)	32 (27.6)	14 (22.2)	55 (28.4)	19 (30.6)	58 (29.4)
Vascular disorders	9 (30.0)	25 (21.6)	18 (28.6)	49 (25.3)	18 (29.0)	59 (29.9)
Renal and urinary disorders	7 (23.3)	19 (16.4)	13 (20.6)	42 (21.6)	15 (24.2)	43 (21.8)
Cardiac disorders	9 (30.0)	16 (13.8)	16 (25.4)	27 (13.9)	10 (16.1)	47 (23.9)
Eye disorders	6 (20.0)	13 (11.2)	9 (14.3)	30 (15.5)	22 (35.5)	47 (23.9)
Injury, poisoning, and procedural complications	7 (23.3)	15 (12.9)	15 (23.8)	18 (9.3)	9 (14.5)	30 (15.2)
Hepatobiliary disorders	4 (13.3)	12 (10.3)	6 (9.5)	17 (8.8)	9 (14.5)	33 (16.8)
Immune system disorders	1 (3.3)	8 (6.9)	5 (7.9)	15 (7.7)	2 (3.2)	23 (11.7)
Neoplasms benign, malignant, and unspecified	3 (10.0)	7 (6.0)	5 (7.9)	14 (7.2)	15 (24.2)	16 (8.1)
Ear and labyrinth disorders	2 (6.7)	5 (4.3)	2 (3.2)	12 (6.2)	2 (3.2)	11 (5.6)

Treatment-emergent AE is an AE starting after the first study drug administration until 28 days after the last dose of the study drug.

*AE* adverse events.

^a^System organ classes with most frequent AEs or AEs of special interest are listed.

^b^Overall: Total number of patients who had treatment-emergent AEs.

Serious AEs (SAEs) were higher in the ≥ 65 years subgroup (76.7%) compared to the < 65 years subgroup (56.9%), although drug-related AEs had a reverse trend (≥ 65 years: 33.3%, < 65 years: 43.1%) (Table 3). The most common drug-related AEs were gastrointestinal disorders (≥ 65 years: 13.3%, < 65 years: 16.4%).

A similar proportion of patients discontinued isavuconazole treatment due to AEs in both subgroups (Table 3) with infection and infestations (≥ 65 years: 6.7%, < 65 years: 4.3%) being the most common. Drug-related AEs leading to permanent discontinuation of isavuconazole were only observed in the < 65 years subgroup (6.0%). A higher proportion AEs leading to death were observed in the ≥ 65 years subgroup (56.7%) than the < 65 years subgroup (23.3%), with infection and infestations (≥ 65 years: 20.0%, < 65 years: 12.9%) being the most common cause.

In SECURE, a similar proportion of AEs was reported in both subgroups of the isavuconazole arm (≥ 65 years: 98.4%, < 65 years: 95.4%), with gastrointestinal disorders (≥ 65 years: 73.0%, < 65 years: 66.0%) being the most common. However, a higher proportion of infections and infestations was reported in the ≥ 65 years subgroup (69.8%) than the < 65 years subgroup (55.7%) in the isavuconazole arm (Table 4); and a higher incidence of psychiatric disorders was reported in the ≥ 65 years subgroup (46.8%) than the < 65 years subgroup (28.9%) in the voriconazole arm.

The proportion of patients with AEs of mild and moderate intensity was lower, and severe intensity was higher in the ≥ 65 years subgroup (mild: 7.9%, moderate: 30.2%, severe: 60.3%) than the < 65 years subgroup (mild: 16.5%, moderate: 27.8%, severe: 51.0%) in the isavuconazole arm. Intensity of AEs was similar in the < 65 years subgroup (mild: 10.2%, moderate: 31.0%, severe: 56.9%) and the ≥ 65 years subgroup (mild: 6.5%, moderate: 33.9%, severe: 59.7%) in the voriconazole arm.

Higher rates SAEs were reported in the ≥ 65 years subgroup than the < 65 years subgroup in the isavuconazole arm (61.9% vs 49.0%) and were similar for both subgroups in the voriconazole arm (58.1% vs 57.4%). Drug-related AEs were reported more frequently in both subgroups of the voriconazole arm (≥ 65 years: 54.8%, < 65 years: 61.4%) than the isavuconazole arm (≥ 65 years: 44.4%, < 65 years: 41.8%) (Table 3). Additionally, in the ≥ 65 years subgroup, a higher proportion of patients in the isavuconazole arm experienced drug-related cardiac disorders (isavuconazole: 6.3%; voriconazole: 0) as well as general disorders and administrative site conditions (9.5% vs 1.6%). In the voriconazole arm, a higher proportion of drug-related psychiatric disorders (isavuconazole: 3.2%; voriconazole: 21.0%), eye disorders (3.2% vs 19.4%), and investigation abnormalities (7.9% vs 14.5%) were reported in the ≥ 65 years subgroup. In the < 65 years subgroup, higher proportions of patients with drug-related AEs were observed only in the voriconazole arm: investigation abnormalities (isavuconazole: 10.3%; voriconazole: 19.3%), hepatobiliary disorders (2.6% vs 11.7%), psychiatric disorders (2.1% vs 8.1%), and eye disorders (3.1% vs 8.1%).

The incidence of permanent drug discontinuation due to AEs was similar in both subgroups (≥ 65 years: 12.7%, < 65 years: 14.9%) with infection and infestations (≥ 65 years: 6.3%, < 65 years: 3.6%) being the most common in isavuconazole arm. However, discontinuations due to AEs were less common in the isavuconazole arm (≥ 65 years: 12.7%, < 65 years: 14.9%) than the voriconazole arm (≥ 65 years: 27.4%, < 65 years: 21.3%) in both subgroups. Drug discontinuation due to drug-related AEs was similar in both subgroups (≥ 65 years: 7.9%, < 65 years: 8.2%) in the isavuconazole arm and were less common among isavuconazole-treated patients than those treated with voriconazole (≥ 65 years: 14.5%, < 65 years: 13.2%) in both subgroups.

The proportion of AEs leading to death was similar between both subgroups of the isavuconazole (≥ 65 years: 27.0%, < 65 years: 23.2%) and voriconazole (≥ 65 years: 30.6%, < 65 years: 26.9%) arms. AEs of infections and infestations were most common in the < 65 years subgroup in both arms (isavuconazole: 8.8% vs voriconazole: 8.6%), although in the ≥ 65 years subgroup infections and infestations were more common in the isavuconazole arm (isavuconazole: 17.5% vs voriconazole: 1.6%) and neoplasms (benign, malignant, and unspecified [including cysts and polyps]) were more common in the voriconazole arm (isavuconazole: 6.3% vs voriconazole: 17.7%).

### Efficacy

In VITAL, all-cause mortality through day 42 after treatment with isavuconazole was higher in the ≥ 65 years than the < 65 years subgroup (30.0% vs 13.8%, Table 5). The overall response at EOT was lower in the ≥ 65 years subgroup than the < 65 years subgroup (27.6% vs 46.8%, Table 6); similar results were noted for clinical response, mycological response, and radiological response at day 42 and EOT (Table 7).

**Table 5:** Analysis of all-cause mortality through Day 42.

Parameter	VITAL study, mITT population		SECURE study, ITT population			
	Isavuconazole		Isavuconazole		Voriconazole	
	≥ 65 years n = 30	< 65 years n = 116	≥ 65 years n = 63	< 65 years n = 195	≥ 65 years n = 62	< 65 years n = 196
All-cause mortality, n (%)	9 (30.0)	16 (13.8)	13 (20.6)	35 (17.9)	14 (22.6)	38 (19.4)
95% CIs	15.29, 50.83	8.47, 22.35	11.47, 32.70	12.83, 24.07	12.93, 34.97	14.10, 25.63
Known deaths	9 (30.0)	14 (12.1)	13 (20.6)	32 (16.4)	14 (22.6)	36 (18.4)
Unknown survival status^a^	0	2 (1.7)	0	3 (1.5)	0	2 (1.0)

Crude mortality rates are calculated within treatment group. The 95% CI for treatment group is based on a binomial distribution.

*CI* confidence interval, *ITT* intent to treat, *mITT* modified ITT, *n* number of patients.

^a^Patients with the last known survival status before day 42 assessment/missing; treated as death(s) while calculating all-cause mortality.

**Table 6:** DRC-assessed overall response at end of treatment (mITT population).

Parameter	VITAL study		SECURE study			
	Isavuconazole		Isavuconazole		Voriconazole	
	≥ 65 years n = 29	< 65 years n = 111	≥ 65 years n = 38	< 65 years n = 105	≥ 65 years n = 25	< 65 years n = 104
Success, n (%)	8 (27.6)	52 (46.8)	9 (23.7)	41 (39.0)	8 (32.0)	39 (37.5)
95% CIs of Success	12.73, 47.25	37.31, 56.55	11.44, 40.24	29.67, 49.06	14.95, 53.50	28.20, 47.53
Complete response	5 (17.2)	20 (18.0)	4 (10.5)	13 (12.4)	3 (12.0)	10 (9.6)
Partial response	3 (10.3)	32 (28.8)	5 (13.2)	28 (26.7)	5 (20.0)	29 (27.9)
Failure^a^, n (%)	21 (72.4)	59 (53.2)	29 (76.3)	64 (61.0)	17 (68.0)	65 (62.5)
Stable	4 (13.8)	27 (24.3)	12 (31.6)	30 (28.6)	8 (32.0)	25 (24.0)
Progression	16 (55.2)	28 (25.2)	17 (44.7)	34 (32.4)	9 (36.0)	40 (38.5)

Crude success rates and exact binomial CIs were calculated within treatment.

Complete and partial response referred to the resolution of attributable clinical symptoms and physical findings.

Failure (no resolution) was defined as no resolution and/or worsening of any attributable clinical symptoms and physical findings.

The 95% CI for treatment group is based on a binomial distribution.

*CI* confidence interval, *DRC* data review committee, *mITT* modified intent to treat, *n* number of patients.

^a^One patient with missing response had an outcome as Failure.

**Table 7:** DRC-assessed clinical response, mycological response, and radiological response (mITT population).

Parameter	VITAL study		SECURE study			
	Isavuconazole		Isavuconazole		Voriconazole	
	≥ 65 years	< 65 years	≥ 65 years	< 65 years	≥ 65 years	< 65 years
Clinical response						
Day 42	n = 29	n = 111	n = 38	n = 101	n = 25	n = 97
Success, n (%)	14 (48.3)	67 (64.4)	21 (55.3)	68 (67.3)	11 (47.8)	58 (59.8)
95% CIs of success	29.45, 67.45	54.43, 73.57	38.30, 71.38	57.28, 76.33	26.82, 69.41	49.35, 69.63
Complete Resolution	4 (13.8)	23 (22.1)	16 (42.1)	42 (41.6)	7 (30.4)	38 (39.2)
Partial Resolution	10 (34.5)	44 (42.3)	5 (13.2)	26 (25.7)	4 (17.4)	20 (20.6)
Failure, n (%)	15 (51.7)	37 (35.6)	17 (44.7)	33 (32.7)	12 (52.2)	39 (40.2)
No resolution	4 (13.8)	10 (9.6)	5 (13.2)	8 (7.9)	2 (8.7)	13 (13.4)
Missing	11 (37.9)	27 (26.0)	12 (31.6)	25 (24.8)	10 (43.5)	26 (26.8)
End of treatment	n = 29	n = 111	n = 38	n = 99	n = 25	n = 96
Success, n (%)	14 (48.3)	67 (64.4)	21 (55.3)	64 (64.6)	13 (52.0)	60 (62.5)
95% CIs of success	29.45, 67.47	54.43, 73.57	38.30, 71.39	54.40, 74.00	31.31, 72.20	52.03, 72.18
Complete resolution	8 (27.6)	38 (36.5)	16 (42.1)	45 (45.5)	10 (40.0)	43 (44.8)
Partial Resolution	6 (20.7)	29 (27.9)	5 (13.2)	19 (19.2)	3 (12.0)	17 (17.7)
Failure, n (%)	15 (51.7)	37 (35.6)	17 (44.7)	35 (35.4)	12 (48.0)	36 (37.5)
No resolution	14 (48.3)	33 (31.7)	17 (44.7)	35 (35.4)	12 (48.0)	36 (37.5)
Missing	1 (3.4)	4 (3.8)	0	0	0	0
Mycological response						
Day 42	n = 29	n = 111	n = 38	n = 105	n = 25	n = 104
Success, n (%)	5 (17.2)	26 (23.4)	11 (28.9)	46 (43.8)	8 (32.0)	43 (41.3)
95% CIs of success	5.85, 35.78	15.96, 32.41	15.43, 45.90	34.14, 53.83	14.95, 53.50	31.77, 51.42
Eradication	0	5 (4.5)	0	0	0	0
Presumed Eradication	5 (17.2)	21 (18.9)	11 (28.9)	46 (43.8)	8 (32.0)	43 (41.3)
Failure	24 (82.8)	85 (76.6)	27 (71.1)	59 (56.2)	17 (68.0)	61 (58.7)
Persistence	1 (3.4)	5 (4.5)	2 (5.3)	4 (3.8)	1 (4.0)	2 (1.9)
Presumed persistence	12 (41.4)	53 (47.7)	13 (34.2)	30 (28.6)	6 (24.0)	33 (31.7)
Missing	11 (37.9)	27 (24.3)	12 (31.6)	25 (23.8)	10 (40.0)	26 (25.0)
End of treatment	n = 29	n = 111	n = 38	n = 105	n = 25	n = 104
Success, n (%)	8 (27.6)	53 (47.7)	9 (23.7)	45 (42.9)	8 (32.0)	45 (43.3)
95% CIs of success	12.73, 47.24	38.18, 57.44	11.44, 40.24	33.24, 52.89	14.95, 53.50	33.59, 53.35
Eradication	1 (3.4)	13 (11.7)	1 (2.6)	1	0	0
Presumed eradication	7 (24.1)	40 (36.0)	8 (21.1)	44 (41.9)	8 (32.0)	45 (43.3)
Failure	21 (72.4)	58 (52.3)	29 (76.3)	60 (57.1)	17 (68.0)	59 (56.7)
Persistence	3 (10.3)	11 (9.9)	6 (15.8)	6 (5.7)	3 (12.0)	10 (9.6)
Presumed persistence	17 (58.6)	43 (38.7)	23 (60.5)	54 (51.4)	14 (56.0)	49 (47.1)
Missing	1 (3.4)	4 (3.6)	0	0	0	0
Radiological response						
Day 42	n = 29	n = 111	n = 38	n = 103	n = 25	n = 103
Success	3 (10.3)	17 (16.3)	9 (23.7)	31 (30.1)	7 (28.0)	37 (35.9)
95% CIs of success	2.19, 27.35	9.82, 24.88	11.44, 40.24	21.45, 39.92	12.07, 49.39	26.70, 45.97
Failure	26 (89.7)	87 (83.7)	29 (76.3)	72 (69.9)	18 (72)	66 (64.1)
Failure to meet success criteria	11 (37.9)	42 (40.4)	15 (39.5)	32 (31.1)	7 (28.0)	38 (36.9)
No post-baseline	4 (13.8)	18 (17.3)	2 (5.3)	15 (14.6)	1 (4.0)	2 (1.9)
Missing	11 (37.9)	37 (34.9)	12 (31.6)	25 (24.3)	10 (40.0)	26 (25.2)
End of treatment	n = 29	n = 111	n = 38	n = 103	n = 24	n = 103
Success	5 (17.2)	35 (34.0)	8 (21.1)	33 (32.0)	8 (33.3)	34 (33.0)
95% CIs of success	5.85, 35.78	16.20, 33.41	9.55, 37.32	23.18, 41.96	15.63, 55.32	24.06, 42.97
Failure	24 (82.8)	79 (76.0)	30 (78.9)	70 (68.0)	16 (66.7)	69 (67.0)
Failure to meet success criteria	18 (62.1)	79 (76.0)	22 (57.9)	47 (45.6)	12 (50.0)	44 (42.7)
No post-baseline	5 (17.2)	18 (17.3)	8 (21.1)	23 (22.3)	4 (16.7)	25 (24.3)
Missing	1 (3.4)	4 (3.8)	0	0	0	0

Crude success rate is calculated within treatment.

Complete and partial response referred to the resolution of attributable clinical symptoms and physical findings.

Failure (no resolution) was defined as no resolution and/or worsening of any attributable clinical symptoms and physical findings.

No post-baseline: No post-baseline radiology available for patients with baseline evidence of radiologic disease.

*CI* confidence interval, *DRC* data review committee, *mITT* modified ITT, *n* number of patients.

In SECURE, all-cause mortality through day 42 was similar between both subgroups and both the isavuconazole (20.6% vs 17.9%) and voriconazole (22.6% vs 19.4%) treatment arms (Table 5). Overall response at EOT was lower in the ≥ 65 years compared to the < 65 years subgroup in the isavuconazole arm (23.7% vs 39.0%) and in the voriconazole arm (32.0% vs 37.5%) (Table 6); similar results were noted for clinical response, mycological response, and radiological response (except in voriconazole arm at EOT) at day 42 and EOT (Table 7). Additionally, overall response at EOT was higher in the voriconazole arm (32.0%) than the isavuconazole arm (23.7%) in the ≥ 65 years subgroup. Moreover, although clinical response at EOT in the ≥ 65 years subgroup was similar in both treatment arms (isavuconazole: 55.3%; voriconazole: 52.0%), the mycological (isavuconazole: 23.7%; voriconazole: 32.0%) and radiological responses (isavuconazole: 21.1%; voriconazole: 33.3%) at EOT were lower in the isavuconazole arm compared to the voriconazole arm.

## Discussion

Currently, available treatment options for IFDs have certain constraints in the older population such as significant DDIs or drug safety and tolerability issues, which may impact their clinical use^19,20^. Therefore, the exploration of the effectiveness of new antifungal agents in older individuals is imperative. Here, we report the findings from a post-hoc analysis of two phase 3 studies of isavuconazole. This analysis indicated, as expected, that the safety and efficacy outcomes were broadly more favorable in the < 65 years compared to the ≥ 65 years subgroup in both studies. However, it must be considered that the number of patients was relatively lower in the ≥ 65 years subgroup in both studies. In addition, the studies were not powered to detect statistical treatment differences between subgroups. In VITAL, all-cause mortality through day 42 was higher in the ≥ 65 years subgroup. In SECURE, all-cause mortality through day 42 was similar between both subgroups and treatment arms, and the safety profile favored isavuconazole over voriconazole in both subgroups.

Aging is an important factor in both drug and dose regimen selection, together with careful monitoring of benefit and risk. Age-associated changes in the biochemical composition of tissues and progressive decrease in physiological capacity may affect the response to a drug^30^, therefore, the safety profile of any therapeutic agent needs to be monitored closely in older individuals. In VITAL, the overall safety profile was more favorable in the < 65 years subgroup compared to the ≥ 65 years subgroup except for drug-related AEs and drug-related AEs leading to permanent discontinuation, which were higher in the < 65 years subgroup. This difference could be attributed to the small sample size (n = 30) in the ≥ 65 years subgroup.

The safety data from the current post-hoc analysis for the SECURE study demonstrated less frequent AEs with isavuconazole treatment compared to voriconazole in both subgroups. Similar to the results of the global analysis^25^, the ≥ 65 years and < 65 years subgroups receiving isavuconazole showed fewer drug-related AEs, as well as AEs and drug-related AEs leading to discontinuation compared with patients receiving voriconazole. Furthermore, drug-related AEs frequently associated with voriconazole such as psychiatric disorders and eye disorders were less common with isavuconazole in the ≥ 65 years subgroup. Similarly, for the < 65 years subgroup, drug-related laboratory abnormalities, hepatobiliary disorders, psychiatric disorders, and eye disorders were also less common in the isavuconazole arm.

Azole antifungals are known to cause hepatocellular injury, thus monitoring of hepatic enzymes is necessitated while receiving treatment^31,32^. Reduced hepatotoxicity with isavuconazole use was reported when compared to other triazoles in the treatment of immunocompromised patients with IFDs^33,34^. Similar to the results of the global analysis^25^, drug-related hepatobiliary AEs in the current analysis were less frequent in the isavuconazole arm (≥ 65 years: 0% vs < 65 years: 2.6%) compared to the voriconazole arm (≥ 65 years: 4.8% vs < 65 years: 11.7%). Other AEs known to be associated with voriconazole (including eye disorders) and discontinuations due to AEs were also less common in the isavuconazole arm compared to the voriconazole arm, consistent with the results of the global analysis^25^.

In this post-hoc analysis, the proportion of AEs with severe intensity was higher in the ≥ 65 years subgroup than the < 65 years subgroup in both studies for the isavuconazole arm. However, the intensity of AEs was similar in the voriconazole arm for both subgroups. Additionally, the proportion of patients with SAEs was noticeably higher in ≥ 65 years subgroup than in the < 65 years subgroup in both studies, probably due to increased comorbidities in the ≥ 65 years subgroup. Furthermore, impaired renal clearance is likely to contribute to an increase in AE incidence in patients with IFDs^35^. Patients with renal impairment were included in VITAL and the proportion of such patients was higher in the ≥ 65 years subgroup compared to the < 65 years subgroup. This may also have contributed to the observed higher proportion of SAEs in the ≥ 65 years subgroup.

Previously, studies assessing mortality in older individuals with IFDs reported a greater prevalence of high-risk factors and worse survival outcomes^14,16,18,22^. In line with these previous studies, data from VITAL revealed that all-cause mortality through day 42 was higher in the ≥ 65 years subgroup than the < 65 years subgroup. In SECURE, all-cause mortality was comparable in the isavuconazole and voriconazole arms, with slightly worse outcomes in the ≥ 65 years subgroup than the < 65 years subgroup.

In VITAL, all efficacy outcomes assessed through EOT were lower in the ≥ 65 years subgroup than the < 65 years subgroup. Clinical response (an individual component of the overall response) was better than the overall response in both subgroups. This may be explained by the observed lower radiological response rates. The rates of mycological and radiological responses, components of overall response, were impacted by missing assessments. Patients with no post-baseline mycological and radiological assessments were conservatively considered failures, and consequently, overall response was assessed as failure in these patients. In SECURE, overall, clinical, and mycological response rates at EOT were lower in the ≥ 65 years than the < 65 years subgroup in both treatment arms. Similar to VITAL, lower overall response compared to clinical response was observed in both subgroups of both treatment arms. The probability of IFDs may be higher in patients whose immune capabilities are more profoundly compromised^36,37^, and underlying disease presentation and subsequent management is more complex. Therefore, it might be reasonable to assume that this would be reflected in poorer treatment outcomes in older individuals.

Since older individuals are usually prescribed several medications and the risk of serious DDIs is more likely^19,38^, it is important to carefully assess the existing drug regimen before adding an azole, to avoid potentially serious and life-threatening DDIs. It is estimated that > 40% of individuals aged ≥ 65 use ≥ 5 medications, and 12% use ≥ 10 different medications^39^. Optimization of antifungal therapy is an urgent need, particularly in the older population experiencing worse disease outcomes and high mortality rates. Although more studies are required, the fact that a lesser degree of interaction observed between isavuconazole and immunosuppressive agents in healthy adults than those reported for other triazole antifungal agents is encouraging^40,41^. Isavuconazole is a valuable addition to antifungal treatment regimens and may be a particularly useful treatment option in older patients with IFDs.

One limitation other than those inherent to any post-hoc analysis was a relatively small sample size for individual age-stratified subgroups, most notably for the ≥ 65 years subgroup in both studies. In addition, the VITAL and SECURE studies were not powered to detect statistical treatment differences between subgroups, and patients enrolled in these controlled trials may not be fully representative of all IFDs affecting older populations. Given the lack of direct clinical evidence for antifungal treatment outcomes in older individuals, real-world studies in older populations may substantiate findings from this study and provide insights into treating IFDs in older patients. Currently, clinicians involved in treating such patients depend solely on pharmacological data for treatment decisions including dose and drug selection.

## Supplementary Information

Supplementary Information.

## Data Availability

The datasets used and/or analyzed during the current study available from the corresponding author on reasonable request.
