# Supplementary material for: Post-hoc analysis of the safety and efficacy of isavuconazole in older patients with invasive fungal disease from the VITAL and SECURE studies
Source: Sci Rep. 2023 Apr 25;13:6730. doi: 10.1038/s41598-023-31788-1 (PMC10127179; doi:10.1038/s41598-023-31788-1)
Supplement: Supplementary file 1 — Supplementary Information. [file 41598_2023_31788_MOESM1_ESM.docx]

**Supplementary information**

**Name and List of ethical committee/Institutional Review Boards who approved the study**

**For VITAL study**:

Institut Jules Bordet, Rue Heger-Bordet 1, 1000 Bruxelles, Belgium; Universitaire Ziekenhuizen Leuven Herestraat 49, 3000 Leuven, Belgium; Cliniques Universitaires de Bruxelles Hopital Erasme Route de Lennik 808, 1070 Bruxelles, Belgium; Hospital de Clinicas da Universidade Federal do Parana, Rua General Carneiro 181 Curitiba-PR, 80060-900 Brazil; Santa Casa de Misericordia de Belo Horizonte Avenida Francisco Sales 1111, Belo Horizonte/MG, Brazil 30150-221; Med. Klinik III-Charite-Universitatsmedizin Berlin Campus Benjamin Franklin Hindenburgdamm 30, 12200 Berlin, Germany; Charite Campus Mitte der Humboldt Universitat Zu Berlin: Med. Klinik und Poliklinik– Oncology/Haematology, Chariteplatz 1; 10117 Berlin, Germany; Leonoren-Apotheke Leonoren-Strasse 97, 12247 Berlin, Germany; Universitatsklinikum Koln Klinik 1 fur Innere Medizin Kerpener Strasse 62, 50937 Koln, Germany; Zentruim fur Innere Medizin (ZIM)-Med. Clinic & Policlinic II, University Medical Center Wuerzburg Division for Infectious Diseases Oberdurrbacher Strasse 6, 97080 Wurzburg, Germany; Apotheke des Universitaetsklinikums Wuerzburg, Apotheke Bereich D2, Josef-Schneider-Str. 2, 97080 Wuerzburg, Germany; Universitaetsklinikum Wuerzburg Zentrum fur Innere Medizin Medizinische Klinik und Polilinik II Haematologie/Onkologie Oberdurrbacher Strasse 6, 97080 Wuerzburg, Germany; Universitaetsklinikum Wuerzburg Medizinische Klinik und Polilinik II Schwerpunkt Infektiologie, Haus C 6 Josef-Schneider-Str. 2, 97080, Wuerzburg, Germany; Deenanath Mangeshkar Hospital & Research Centre Erandawane, Pune 411004, India; Rambam Medical Center Aliya st. Bat Galim, POB 9602 Haifa 31096, Israel; Infectious Diseases Unit Chaim Sheba Medical Center Tel Hashomer Ramat Gan 5262000, Israel; Tel Aviv Sourasky Medical Center 6 Weizman St Tel Aviv 6423906, Israel; American University of Beirut Medical Center PO Box 11-0236, Riad El Solh Beirut, Lebanon 1107 2020; Av. Francisco I. Madero Pte.S/N Edificio Barragan, 3 er piso, Col. Mitras Centro Monterrey Nuevo Leon, Mexico; Hospital Universitario “Dr. Jose Eleuterio Gonzalez” Centro de Investigacion, Prevencion y Tratamiento de Infecciones Respiratorias, Av. Francisco I. Madero Pte.S/N, Edificio Barragan, 3 er piso, Col. Mitras Centro, Monterrey Nuevo Leon, Mexico; Hospital Universitario “Dr. Jose Eleuterio Gonzalez” de la Universidad Autonoma de Nuevo Leon Avenida Francisco I. Madero y Gonzalitos S/N, Col. Mitras Centro, Monterrey Nuevo Leon, Mexico 64040; State Institution “Hematology Research Centre RAMS”, 4a. Novy Zykovsky proezd Moscow, 125167, Russia; The Catholic University of Korea Seoul St. Mary’s Hospital, 222 Banpo-Daero Seocho-gu, Seoul 137-701, South Korea; The Catholic University of Korea Seoul St. Mary’s Hospital, 505 Banpo-dong, Seocho-gu, Seoul 137-701, South Korea; St. Mary’s Hospital, 62 Yeouido-dong, Yeongdeungpo-gu Seoul, 150-713, South Korea; Samsung Medical Center 81 Irwon-ro Gangnam-Gu, Seoul, 135-710, South Korea; Samsung Medical Center, 50 Irwon-Dong Gangnam-Gu Seoul, 135-710, South Korea; Department of Internal Medicine Faculty of Medicine, Chiang Mai University, 110 Intavaroros Road, Amphoe Muang Chiang Mai, 50200, Thailand; Division of Infectious Disease and Tropical Medicine Department of Medicine, Faculty of Medicine Srinagarind Hospital, Khon Kaen University, Khon Kaen 40002, Thailand;

Division of Clinical Pharmacology, Department of Medicine, Faculty of Medicine, Songklanagarind Hospital Prince of Songkla University, 15 Karnjanavanit Road, Hat Yai, Songkla Province, 90110 Thailand; Division of Infectious Disease Department of Medicine, Maharat Nakon Ratchasima Hospital 49 Chang Phueak Road, Mueang, Nakhonratchasima, 30000 Thailand; University of Alabama at Birmingham 619 19^th^ St. South, Birmingham, Alabama 35249; Infectious Disease Clinic 908 20^th^ Street South, Birmingham, Alabama 35294, USA; The Kirklin Clinic 2000 6^th^ Avenue South, Birmingham, Alabama 35233, USA; Henry Ford Health System 2799 W. Grand Blvd., Detroit, Michigan 48202, USA; Ochsner Clinic Foundation 1514 Jefferson Highway, New Orleans, Louisiana 70121, USA; Regional Infectious Disease & Infusion Center, Inc. 830 W. High Street, Suite 255 Lima, Ohio 45801, USA; St. Rita’s Medical Center 730 W. Market Street Lima, Ohio, 45801, USA; Triumph Hospital-Lima 730 W. Market Street 6^th^ Floor, Lima, Ohio 45801, USA; Brigham and Women’s Hospital 75 Francis Street, Boston, MA 02115, USA; The University of Texas MD Anderson Cancer Center (UTMDACC) Infectious Diseases Unit 1460 1515 Holcombe Blvd, Houston, Texas 77030, USA; Emory University, 101 Woodruff Circle, WMB Suite 2101, Atlanta, Georgia 30322, USA; Emory University Hospital 1364 Clifton Road, NE Atlanta, Georgia, 30322, USA; The Emory Clinic,1365 Clifton Road NE Atlanta, Georgia 30322, USA; Emory University Investigational Drug Services, The Emory Clinic Bldg. A, Suite 1200 1365 Clifton Road, NE Atlanta, Georgia 30322, USA; Emory University, 101 Woodruff Circle, Suite 1304 Atlanta, Georgia 30322, USA; UC Davis Medical Center 2315 Stockton Blvd., Sacramento, California 95817, USA; University of Minnesota Center, Fairview 420 Delaware Street SE, Minneapolis, Minnesota 55455, USA; Fred Hutchinson Cancer Research Center 1100 Fairview Ave. North, Seattle, Washington 98109-1024, USA; Seattle Cancer Care Alliance (SCCA) 825 Eastlake Ave. E, Seattle, Washington 98109-1023, USA; University of Washington Medical Center 1959 NE Pacific Street, Seattle, Washington 98195, USA; UPMC, 200 Lothrop Street PGH, PA 15213, USA; Stanford Hospital School of Medicine Division of Bone Marrow and Transplantation Program, 300 Pasteur Drive, Stanford, California 94305, USA; University of Chicago Hospitals 5841 S. Maryland Ave., Chicago, IL 60637, USA.

**For SECURE study**

Sanatorio de la Trinidad Mitre, Bartolome Mitre 2553 (C1039AAO), Buenos Aires, Argentina; Instituto Medico Especializado Alexander Fleming, Cramer 1180 (1426) CABA, Buenos Aires, Argentina; Sanatorio Güemes, Francisco Acuña de Figueroa 1240, (C1180AAX) Ciudad Autonoma de Buenos Aires, Argentina; Hospital General de Agudos Dr. Cosme Argerich, Almirante Brown 240, (C1155ADP) Ciudad Autonoma de Buenos Aires, Argentina; Hospital General de Agudos Dr. Carlos G. Durand, Av. Díaz Velez 5044, (C1405DCR) Ciudad Autonoma de Buenos Aires, Argentina; Infection Management Services Building 17, Level 1, Princess Alexandra Hospital Ipswich Road, Woolloongabba, Brisbane, Queensland 4102, Australia; (HOCA) @ Mater Level 5, Mater Medical Centre 293 Vulture Street, South Brisbane, QLD 4101, Australia; Mater Private Hospital Brisbane 301 Vulture Street, South Brisbane, QLD 4101, Australia; Mater Private Cardiology Suite 10, Level 6, Mater Medical Center 293 Vulture Street, South Brisbane, QLD 4101, Australia; Haematology Department Level 2 North Block, Royal Perth Hospital Wellington Street, Perth, Western Australia 6001, Australia; Institut Jules Bordet Rue Héger-Bordet 1, 1000, Bruxelles, Belgium; Universitair Ziekenhuis Gent De Pintelaan 185, 9000, Gent, Belgium; Universitaire Ziekenhuizen Leuven Herestraat 49, 3000, Leuven, Belgium; Algemeen Ziekenhuis Sint-Jan Brugge-Oosterde AV Campus St-Jan, Ruddershove 10, 8000, Brugge, Belgium; Cliniques Universitaires de Bruxelles Hopital Erasme Route de Lennik 808, 1070, Bruxelles, Belgium; Hospital Universitario Clementino Fraga Filho-UFRJ Rua Professor Rodolpho Paulo Rocco 255, Ilha do Fundao, Cidade Universitaria, 5º andar UPS Rio de Janeiro/RJ, 21941-913, Brazil; Hospital de Clinicas da Universidad Federal do Parana Rua General Carneiro 181, Curitiba-PR, 80060-900, Brazil; Hospital das Clinicas da Faculdade de Medicina de Ribeirao Preto da Universidade de São Paulo (HCFMRP-USP), Avenida Bandeirantes 3900 Campus Universitario Monte Alegre, Ribeirao Preto-SP, 14048-900, Brazil; Hospital das Clinicas da Faculdade de Medicina de Ribeirao Preto da Universidade de São Paulo (HCFMRP-USP), Avenida Bandeirantes 3900 Campus Universitario Monte Alegre Ribeirao Preto-SP, 14048-900, Brazil; Hospital Nossa Senhora das Gracas Rue Alcides Munhoz 433, Curitiba-PR, CEP 80810-040, Brazil; Centro Medico São Francisco, Rua Desembargador Vieira Cavalcanti 1089 Curitiba-PR, CEP 80810-050, Brazil; Hospital Felicio Rocho Avenida do Contorno 9530, Belo Horizonte-MG, 30110-943, Brazil; Santa Casa de Misericordia de Belo Horizonte Avenido Francisco Sales 1111, Belo Horizonte-MG, 30150-221, Brazil; Ottawa Hospital – General Campus 501 Smyth Road, Ottawa, ON K1H 8L6, Canada; Hamilton Health Sciences - Juravinski Hospital 711 Concession Street, Room 500, Section M Hamilton, ON L8V 1C3, Canada; Hospital Base Valdivia Av. Simpson 850 Valdivida, Chile; Hospital Dr. Hernan Henriquez Aravena Manual Montt 115, Temuco, Chile; Hospital Dr. Hernan Henriquez Aravena Av. Prieto Norte 248, Temuco, Chile; Hospital Dr. Sotero del Rio, Av. Concha y Toro 3459, Puente Alto, Santiago, Chile; Department of Infectious Diseases, Huashan Hospital Fudan University No. 12 Urumuqi Middle Road Shanghai 200040, China; Department of Hematology Changhai Hospital of Shanghai No. 168 Changhai Road Yangpu District, Shanghai 200433, China; Department of Hematology Peking University Third Hospital No. 49 North Garden Road Haidian District, Beijing 100083, China; Department of Hematology Peking University Third Hospital No. 10 Chedaogou, Haidian District, Beijing 100089, China; Department of Hematology, The People’s Hospital of Guangxi Zhuang Autonomous Region, No. 6 Taoyuan Road, Nanning, Guangxi Province 530021, China; Oncology Center The First Hospital of Jilin University No. 71 Xinmin Street Changchun, Jilin Province 130021, China; Department of Hematology, West China Hospital Sichuan University, No. 37 Guoxue Xiang, Chengdu Sichuan Province 610041, China; Department of Hematology, The Affiliated Union Hospital of Fujian Medical University, No. 29 Xin Quan Road, Fuzhou, Fujian Province 350001, China; Department of Transplant, The Third Xiangya Hospital of Central South University No. 138 Tongzipo Road, Changsha, Hunan Province 410013, China; Department of Hematology, 1st Affiliated Hospital of Zhejiang University, Medical School, No. 79 Qingchun Road, Hangzhou, Zhejiang Province 310003, China; Department of Hematology, The First Affiliated Hospital of Nanjing Medical University, No. 300 Guangzhou Road, Nanjing, Jiangsu Province 210029, China; Alexandria University Hospital - Clinical Research Center 17 Champlion Street, El Messalah Square Alexandria 21131, Egypt; National Cancer Institute Kasr El Aini Street, Fom El Khalig Square Cairo 11796, Egypt; Nasser Institute Hospital 1351 Kornish El Nile Street, Shobra-Aghakhan, Cairo 12655, Egypt; Hôpital Hautepierre, Department d’Hematologie et Oncologie 1 Avenue Moliére, 67098 Strasbourg, France; Hopital Civil, 1 place de l’hopital 67091 Strasbourg, France; Service d’Hematologie Clinique CHU de Dijon – Hôpital d’Enfants, 10 Boulevard Marechal de Lattre de Tassigny 21000, Dijon, France; Service d’Hematologie Clinique CHU de Dijon Hopital du Bocage, 2 boulevard Marechal de lattre de Tassigny 21000, Dijon, France; CHU de Nantes- Hotel Dieu Service d’Hématologie Clinique Place Alexis Ricordeau, 44093, Nantes Cedex 01, France; Department of Surgery University of Schleswig-Holstein Campus Luebeck, Ratzeburger Allee 160, 23538, Luebeck, Germany; Charité Universitatsmedizin Berlin – Campus Charité Mitte, Charité Campus Benjamin Franklin-Haematology and Oncology, Hindenburgdamm 30, 12200, Berlin, Germany; Herzzentrum Universitat Leipzig Struempellstrasse 39, 04289, Leipzig, Germany; Universitatsklinikum Koln Klinik 1 fur Innere Medizin Kerpener Strasse 62, 50937, Koln, Germany; Zentrum fur Innere Medizin (ZIM)-Med. Clinic & Policlinic II, University Medical Center Wuerzburg Division for Infectious Diseases Oberdurrbacher Strasse 6, 97080, Wurzburg, Germany; Stadtisches Krankenhaus Munchen Neuperlach Klinik fur Onkologie und Hamatologie, Oskar-Maria-Graf-Ring 51, 81737, Munchen, Germany; University Medical Center Aachen, Dept. Oncology/Hematology, Medicine IV Pauwelstrasse 30, 52057, Aachen, Germany; St. Istvan and St. Laszlo Hospital Stem Cell Transplantation Unit Gyali u. 5-7, H-1097, Budapest, Hungary; Borbenyi: Szegedi Tudomanyegyetem, AOK Szent-Gyorgyi Albert, Orvostudomanyi es Gyogyszeresztudomanyi Centrum, II. sz. Belgyogyaszati Klinika es Kardiologiai Kozpont H-6720 Szeged, Koranyi fasor 6, Hungary; Petz Aladar Megyei Oktato Korhaz, Il. Belgyogyaszat es Haematologiai Reszleg 9024 Gyor, Vasvari P. u. 2-4, Hungary; Tata Memorial Hospital Dr. Ernest Borges Marg, Parel, Mumbai 400012, India; Kasturba Medical College (KMC) Hospital Attavar, N G Road, Mangalore 575001 Karnataka, India; Metro Multispeciality Hospital L-94 Sector 11, Noida 201301, Uttar Pradesh, India; Nizam’s Institute of Medical Sciences, Punjagutta, Hyderabad 500082 Andhra Pradesh, India; Apollo Hospitals Jubilee Hills Hyderabad 500096, Andhra Pradesh, India; Kasturba Hospital Madhav Nagar Manipal 576104 Karnataka, India; Sahyadri Specialty Hospital 30C Erandawane, Karve Road, Pune 411004, India; Deenanath Mangeshkar Hospital & Research Centre Erandawane, Pune 411004, India; Hadassah University Hospital – Ein Kerem Kiryat Hadassah, POB 12000, Jerusalem 9112001, Israel; Rambam Medical Center, Aliya St., Bat Galim, POB 9602 Haifa 3109601, Israel; Infectious Diseases Unit Chaim Sheba Medical Center Tel Hashomer, Ramat Gan 5262000, Israel; Tel Aviv Sourasky Medical Center 6 Weizman St., Tel Aviv 6423906, Israel; Rabin Medical Center – Beilinson Hospital 39 Jabutinski St., Petah Tikva 4941492, Israel; IRCCS Policlinico San Matteo, Clinica Ematologica, Viale Golgi, 19, 27100, Pavia, Italy; Divisione di Ematologia, A.O. Ospedale Niguarda Ca’ Granda, Piazza Ospedale Maggiore n.3 20162, Milano, Italy; University Malaya Medical Centre Jalan Universiti, 59100, Kuala Lumpur, Malaysia; Hospital Ampang, Jalan Mewah Utara Pandan Mewah, 68000, Ampang, Selangor Darul Ehsan, Malaysia; Hospital Universitario “Dr. Jose Eleuterio Gonzalez” de la Universidad Autonoma de Nuevo, Leon Avenida Francisco I. Madero y Gonzalitos S/N, Col. Mitras Centro C.P. 64040 Monterrey, Neuvo Leon, Mexico; Hospital Universitario “Dr. Jose Eleuterio Gonzalez” Centro de Investigacion, Prevencion y Tratamiento de Infecciones Respiratorias, Av. Francisco I. Madero Pte S/N, Edificio Barragan 3er piso, Col. Mitras Centro C.P. 64040 Monterrey, NL, Mexico; University Medical Center St. Radboud Geert Grooteplein - Zuid 10, 6525, GA Nijmegen, The Netherlands; Palmerston North Hospital Ruahine Street, Palmerston North 4442, New Zealand; Samodzielny Publiczny Centralny Szptial Kliniczny Klinika Chorob Wewnetrznych, ematologii I Onkologii ul. Banacha 1, 02-097, Warszawa, Poland; State Budget Educational Institution of High Professional Education “North-West State Medical University named after I. I. echnikov” of Ministry of healthcare and social development of Russian Federation 1/28 Santiago de Cuba str., St. Petersburg 194291, Russia; State Educational Institution of Additional Professional Education “Saint-Petersburg Medical Academy of Postgraduate Education of RosZdrav 1/28 Santiago de Cuba str. St. Petersburg 194291, Russia; Public Educational Institution of additional professional education “Saint-Petersburg Medical Academy of Postgraduate Education of Russia Health Care” Department of Clinical Mycology, Allergology and Immunology, 1/28 Santiago-de-Cuba str. St. Petersburg 194291, Russia; State Institution “Hematology Research Centre RAMS” 4a Novy Zykovsky proezd, Moscow 125167, Russia; Public Educational Institution of High Professional Education “Saint Petersburg State Medical University of Russian Health Care named after I.P. Pavlov”, Clinic of Bone Marrow transplantation 12 Rentgena str., St. Petersburg 197089, Russia; State Healthcare Budget Institution “Leningrad Regional Clinical Hospital” 45-49 Lunacharskogo pr., St. Petersburg 194291, Russia; State Institution “Russian Oncology Research Centre named after N.N. Blokhin RAMS”, 24 Kashirskoye shosse, Moscow 115478, Russia; State Healthcare Institution, “Republican Hospital named after V. A. Baranov” 3 Pirogova st., Petrozavodsk 185019, Russia; The Catholic University of Korea Seoul St. Mary’s Hospital, 222 Banpo-Daero, Seocho-gu Seoul 137-701, South Korea; St. Mary’s Hospital 62 Yeouido-dong Yeungdeungpo-gu, Seoul 150-713, South Korea; Severance Hospital, Yonsei University Health System 50 Yonsei-ro, Seodaemun-gu Seoul 120-752, South Korea; Asan Medical Center, 88 Olympic-ro, 43 gil, Songpa-gu, Seoul 138-736, South Korea; Samsung Medical Center 81 Irwon-ro, Gangnam-Gu, Seoul 135-710, South Korea; Samsung Medical Center, 50 Irwon-Dong, Gangnam-Gu Seoul 135-710, South Korea; Gachon University Gil Hospital 1198 Guwol-dong, Namdong-gu Incheon 405-760, South Korea; Hospital Universitario de Salamanca Paseo de San Vicente, 58-182 37007, Salamanca, Spain; : Klinik fur Infektionskrankheiten und Spitalhygiene Universitaetsspital Zuerich, CH-8091, Zurich, Switzerland; Klinik fur Infektionskrankheiten und Spitalhygiene Abteilung fur Spitalhygiene, Universitatsspital Zurich Haldenbachstrasse 14C, CH-8091, Zurich, Switzerland; Department of Medicine Faculty of Medicine, Siriraj Hospital, Mahidol University 2 Prannok Road, Bangkok-noi Bangkok 10700, Thailand; Division of Infectious Disease and Tropical Medicine, Department of Medicine, Faculty of Medicine Siriraj Hospital, Mahidol University, Bangkok 10700, Thailand; Department of Internal Medicine, Faculty of Medicine Chiang Mai University, 110 Intavaroros Road, Amphoe Muang Chiang Mai 50200, Thailand; Section of Infectious Disease, Department of Medicine, Faculty of Medicine Chiang Mai University, Chiang Mai 50200, Thailand; Division of Infectious Disease and Tropical Medicine Department of Medicine, Faculty of Medicine Srinagarind Hospital, Khon Kaen University, Khon Kaen 40002, Thailand; Division of Clinical Pharmacology, Dept of Medicine, Faculty of Medicine,Songklanagarind Hospital, Prince of Songkla University 15 Karnjanavanit Road, Hat Yai, Songkla Province 90110, Thailand; Division of Infectious Disease, Department of Medicine Maharat Nakhon Ratchasima Hospital, 49 Chang Phueak Road, Mueang, Nakhonratchasima 30000, Thailand; Marmara University Medical Faculty, Department of Internal Medicine Infectious Disease Division, Mimar Sinan cad. No:41 Ust Kaynarca Fevzi Cakmak Mah. Pendik/Istanbul, Marmara University Medical Faculty, Department of Internal Medicine Infectious Disease Division, Tophanelioglu Cad No: 13 Kat 1 34662, Altunizade Istanbul, Turkey; New York Presbyterian Hospital Weill Cornell Medical College 525 East 68th Street, New York, NY 10065, USA; University of Alabama at Birmingham 619 19th St. So., Birmingham, AL 35294, USA; Infectious Diseases Clinic 908 20th Street So., Birmingham, AL 35294, USA; The Kirklin Clinic 2000 6th Ave. So., Birmingham, AL 35233, USA; Regional Infection Diseases & Infusion Center Inc. 830 W. High Street, Suite 255, Lima, Ohio 45801, USA; St. Rita’s Medical Center 730 W. Market St., Lima, Ohio 45801, USA; Triumph Hospital-Lima, 730 W. Market St., 6th Floor Lima, Ohio 45801, USA; Infectious Disease of Indiana, PSC 11455 N. Meridian St. Suite 200 Carmel, Indiana 46032, USA; St. Vincent Hospital & Health Care Center 2001 W. 86th Street, Indianapolis, IN 46260, USA; St. Vincent Hospital & Health Care Center 13500 N. Meridian Street, Carmel, Indiana 46032, USA; Brigham and Women’s Hospital, 75 Francis Street, Boston, MA 02115, USA; MD Anderson Cancer Center 1515 Holcombe Blvd., Houston, TX 77030, USA; St. John’s Hospital, 800 East Carpenter Street Springfield, IL 62702, USA; Memorial Medical Center 701 North First Street Springfield, IL 62702, USA; Springfield Clinic, Infectious Diseases St. John’s Pavilion, 301 N. Eighth Street, 1st Floor Springfield, IL 62701, USA; Springfield Clinic, Department of Research 1130 S. 6th St., Suite 100, Springfield, IL 62703, USA; Springfield Clinic Research Department Main Campus-West Building, Suite 4300 1025 South Sixth St., Springfield, IL 62703, USA; Springfield Clinic Research Department, 326 North Seventh St., Rm 202 Springfield, IL. 62701, USA; Albany Medical Center Hospital 43 New Scotland Ave., Albany, NY 12208, USA; Albany Medical College 47 New Scotland Ave. Albany, NY 12208, USA; Albany Medical Center Hospital 43 New Scotland Ave., Albany, NY 12208, USA; Albany Medical College 47 New Scotland Ave. Albany, NY 12208, USA; UCSF Medical Center 505 Parnassus Ave., San Francisco, CA 94143, USA; UCSF Medical Center 500 Parnassus Ave, San Francisco, CA 94143, USA; UCSF Medical Center Department of Medicine, Division of Infectious Diseases, 513 Parnassus Avenue, Rm S-410 San Francisco, CA 94143, USA; University of Chicago Hospitals 5841 South Maryland Avenue, Chicago, IL 60637, USA; UMass Memorial Medical Center University Campus, 55 Lake Ave. North Worcester, MA 01655, USA; UMass Memorial Medical Center Memorial Campus, 119 Belmont St., Worcester, MA 01605, USA; University of Kansas Cancer Center and Medical Pavilion 2330 Shawnee Mission Parkway, Westwood, KS 66205, USA; University of Kansas Medical Center 3901 Rainbow Blvd., Kansas City, KS 66160, USA.
